# Developing Mammary Gland Models for Biomedical Applications

**DOI:** 10.34133/research.1240

**Published:** 2026-04-09

**Authors:** Junqi Zhao, Xiang Lin, Hui Zhang, Yuanjin Zhao

**Affiliations:** ^1^ Joint Centre of Translational Medicine, Wenzhou Institute of University of Chinese Academy of Sciences, The First Affiliated Hospital of Wenzhou Medical University, Wenzhou 325035, China.; ^2^ Department of Rheumatology and Immunology, Nanjing Drum Tower Hospital, School of Biological Science and Medical Engineering, Southeast University, Nanjing 210096, China.; ^3^ Co-innovation Center of Neuroregeneration, Nantong University, Nantong 226001, China.

## Abstract

A comprehensive understanding of the physiological functions of mammary glands and the mechanisms underlying breast diseases is crucial for promoting women’s health and well-being. This review systematically summarizes the development and applications of in vitro mammary gland models, focusing on key components including cell types, hydrogel matrix, and breast-on-a-chip platforms. We begin with a detailed overview of primary mammary epithelial cells, various cell lines, organoids, and multicellular coculture systems, highlighting their respective characteristics and advantages in mimicking mammary gland physiology and pathology. Next, we examine the essential role of hydrogel matrix in providing extracellular matrix support and facilitating cellular interactions. Furthermore, recent advances in breast-on-a-chip technologies are reviewed, covering their design, fabrication, and functional capabilities that enable high-throughput drug screening and disease mechanism investigations. Finally, we explore the applications of these in vitro models in mammary gland development, breast cancer research, and drug discovery, underscoring their critical role in improving prevention and treatment strategies. By integrating these state-of-the-art technologies, this review provides researchers with a comprehensive perspective to better understand and apply in vitro mammary models in innovative studies.

## Introduction

The mammary gland is an essential female organ specialized for milk synthesis and secretion. Beyond providing nutritional support that promotes neonatal growth and development, the mammary gland delivers immunoprotective bioactives in milk that bolster infant immunity [1–3]. However, it is also the primary site of breast cancer—a highly prevalent and life-threatening malignant tumor affecting women globally [4–7]. Breast cancer not only endangers patients’ survival but also imposes considerable psychological distress and social burden [8]. Therefore, understanding the biological characteristics of the mammary gland and the mechanisms underlying its physiological dynamics is crucial for the prevention, early diagnosis, and treatment of breast-related diseases. In particular, elucidating the pathogenesis of breast cancer can help identify novel targets for more effective therapies, thereby improving patients’ overall survival and quality of life. To gain deeper insights into mammary physiology and pathology, precise mammary gland models are urgently needed.

The mammary gland is a dynamic organ, which undergoes a complex development process spanning embryogenesis, puberty, sexual maturity, and the pregnancy–lactation cycle [9,10]. This process is tightly regulated by various hormones, including estrogen, progesterone, prolactin, and oxytocin, which orchestrate both the structural maturation of the gland and the synthesis and secretion of milk [2,11]. In addition to hormone control, mammary gland physiology is shaped by genetic factors, environmental exposures, and lifestyle influences [12]. The mammary gland’s physiological complexity is simply too complex for traditional 2-dimensional (2D) cell culture models, rendering them inadequate for studying its development and pathology. To bridge this gap, sophisticated models like 3D organoids and coculture system have emerged, offering more physiologically relevant environments. Engineered hydrogels have been developed to encapsulate cells while mimicking the biochemical and biomechanical properties of native tissues. Building on these advances, mammary gland-on-a-chip technology has further integrated cells and extracellular matrix (ECM) with microengineered platforms to better recapitulate the gland’s architecture and dynamic function. Collectively, these innovative models provide powerful tools for investigating the interplay between mammary epithelial cells (MECs) and their stroma, as well as their responses to external stimuli, thereby accelerating research into both normal physiology and pathology.

**Fig. 1.**
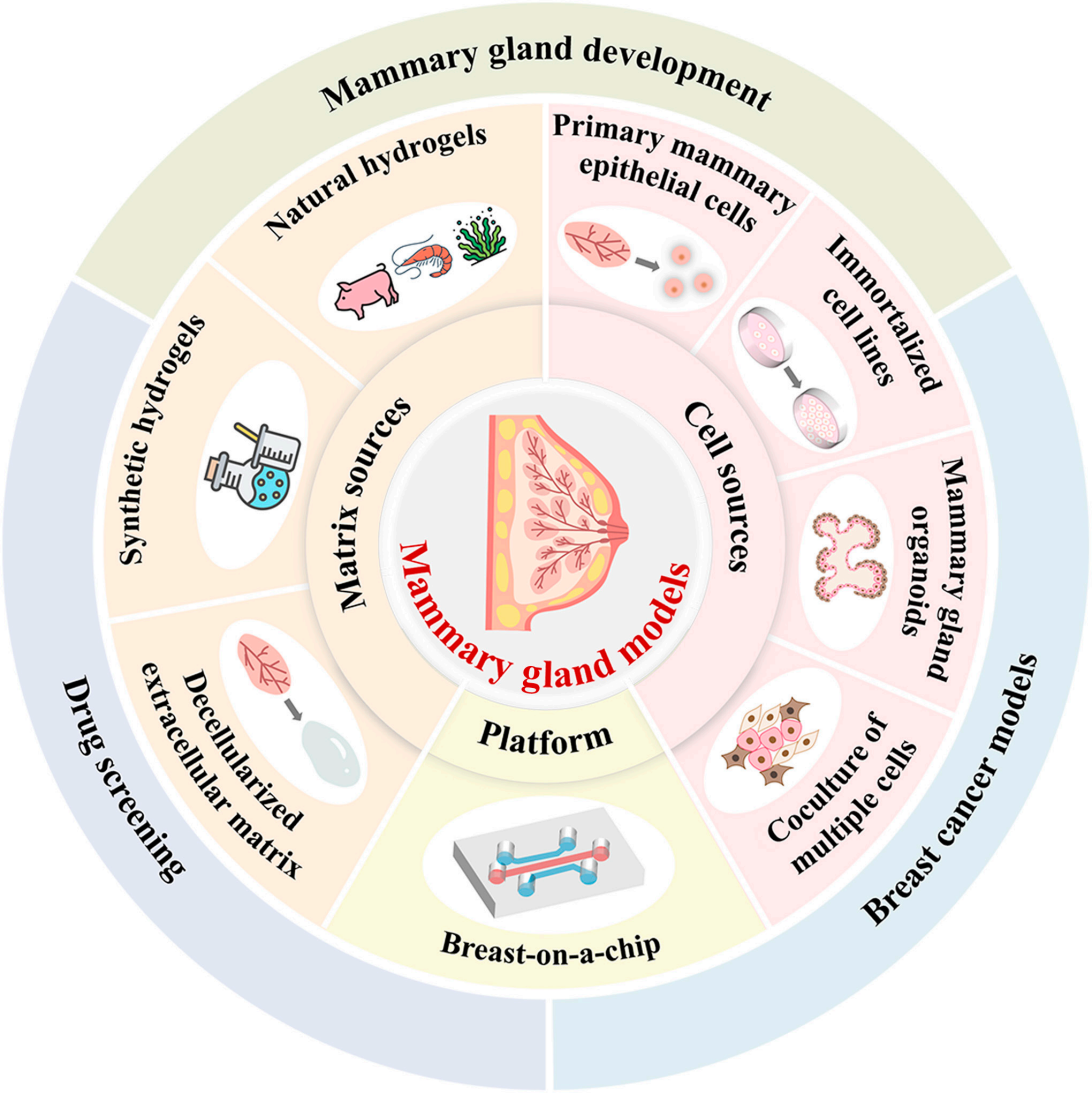
Schematic illustration of various mammary gland models and their biomedical applications. Some icons designed by Freepik (www.freepik.com).

Building on these recent advancements, this review aims to comprehensively summarize the development and application of mammary gland models (Fig. 1). First, we outline various cell-based models, including those derived from primary cells, cell lines, organoids, and coculture systems (Table 1). Next, we discuss hydrogel materials used for cell encapsulation, ranging from natural matrices to synthetic polymers and decellularized extracellular matrix (dECM) (Table 2). We then explore the design and utility of emerging breast-on-a-chip platforms for simulating breast tissue within a microengineered environment. Furthermore, we highlight the biomedical applications of these models in mammary gland development studies, breast tumor modeling, and drug screening. Finally, we address current challenges in enhancing model fidelity and complexity and emphasize future directions toward clinical translation and personalized medicine.

**Table 1.** Comparison of different cell sources in in vitro model systems. (+) low/limited; (++) medium/partial; (+++) high/full.

Feature	PMECs	iPSC-MECs	Cell lines	MGOs	Multicellular coculture ^a^
Success rate of establishment	++	+	+++	+	++
Genetic stability	+++	++	+ ^b^/++ ^c^	+++	++
Long-term maintenance	+	++	+++	+	+
Relative cost	++	++	+	+++	++
Morphological complexity	++	++	+	+++	+++
Recapitulation of functions	++	++	+	+++	+++
High-throughput screening	+	++	+++	++	+

^a^ Multicellular coculture includes cell line–stromal and organoid–stromal coculture systems. Values indicate composite performance across these strategies; refer to Cell lines and MGOs columns for specific characteristics.

^b^ Cancer cell lines.

^c^ Normal cell lines.

**Table 2.** Comparison of different hydrogels for mammary gland modeling

Category	Characteristic	Hydrogel	Properties	Application	Reference
Natural matrices	Animal/plant-derived; bioactive; substantial batch variability	Laminin	Major basement membrane component essential for cell adhesion and polarization	Induces apical–basal polarity of MECs	Grafton et al. [59]
		Collagen I (outer) + Matrigel (inner) bilayer	Reconstituted basement membrane-like structure	Guides MECs to form a polarized monolayer	Bischel et al. [60]
		Spatially modified collagen I	Treated to reduce inter-fiber spacing	Promotes endogenous laminin deposition and stable cell polarity	Cho et al. [61]
		Matrigel	Soluble basement membrane extract containing laminin, collagen IV, and growth factors	Supports cyst-like MGO formation with clear lumens but limited branching	Nguyen-Ngoc et al. [62]
		Collagen I	The most abundant ECM component in the mammary fat pad	Supports branched MGOs; branch ends form alveolar structures in suspension gels or cylindrical structures in attached gels	Fernández et al. [67]
		Collagen I + Matrigel hybrid	Composite hydrogel combining structural and bioactive cues	Supports MGOs with optimal polarity establishment	Yuan et al. [41]
		Alginate	Ionically crosslinked hydrogel; suitable for cell encapsulation	Enables efficient encapsulation of breast tumor fragments in microfluidic systems to culture tumor organoids for drug screening	Fang et al. [68]
Synthetic matrices	Chemically defined; highly tunable; inherently bioinert	PLGA	Good biocompatibility and biodegradability	Allows 3D culture of MCF-7 cells on porous PLGA microspheres for tumor xenograft construction in mice	Kang and Bae [70]
		PEG	Bioinert with widely tunable mechanical properties	Cell adhesive peptide-functionalized PEG hydrogels enable breast cancer organoid culture with defined matrix stiffness.	Bock et al. [71]
		PIC	Thermoreversible physical gelation; long-term structural stability; tunable mechanics	RGD-functionalized PIC hydrogels support the differentiation of mammary fragments or purified MECs into organoids and maintain their branching capacity long-term.	Zhang et al. [72]
		SAPHs	Artificially designed self-assembling short peptides; tunable mechanical properties and degradation profiles	PeptiGel SAPHs with different charges mimic the mammary microenvironment for 3D culture of MECs.	Lingard et al. [73]
dECM	Tissue/cell-derived ECM; tissue-specific; complex composition	Decellularized scaffold	Preserved intact ECM architecture; high mechanical strength	Direct cell seeding for in vitro breast cancer modeling	Wishart et al. [77] Jin et al. [78]
		Decellularized hydrogel	Injectability; shape adaptability	Functions as a bioink for 3D bioprinting of breast tumor models	Mollica et al. [79] Shukla et al. [82]

## Cell Sources for Mammary Gland Models

### Primary mammary epithelial cells

Primary mammary epithelial cells (PMECs) are directly isolated from living organisms or fresh milk and cultured in vitro [13]. When maintained in short-term 2D monolayer cultures, they preserve the tissue-specific characteristics and biological authenticity of their origin, making them ideal models for investigating physiological and pathological processes as well as drug responses [14,15]. PMECs derived from healthy mammary glands provide a valuable model for studying normal mammary physiology. For instance, La Mantia et al. [16] utilized human PMECs to construct a blood–milk barrier model for evaluating drug safety during breastfeeding. Conversely, PMECs obtained from patient tumors largely retain the genetic profile of the original malignancy [17]. This is particularly important as it allows researchers to explore genetic and phenotypic heterogeneity within tumors, which can inform targeted therapies and personalized medicine approaches [17,18].

Despite these advantages, a prolonged culture of PMECs on 2D substrates disrupts physiological cell–cell and cell–matrix crosstalk, leading to alterations in cellular signaling pathways and a progressive loss of in vivo-like properties [19–21]. Consequently, the finite replicative capacity of primary cells restricts their broader applicability. The in vivo developmental process from embryonic stem cells to non-neural ectoderm and then to mammary tissue can be recapitulated in vitro. Induced pluripotent stem cells (iPSCs) can be directed to differentiate into mammary-like cells, which are highly similar to PMECs at the cellular, transcriptional, and functional levels, making them an ideal substitute. Currently, only one case of iPSC-MEC-related mammary models has been reported, and its induction cycle takes as long as 40 d [22]. Therefore, research on the induction and application of iPSCs-MECs remains an area worthy of exploration.

### Immortalized cell lines

MCF-10A cell line, derived from mammary tissue of a patient with fibrocystic breast disease, is the most widely used model of normal MECs. MCF-10A cells are used in in vitro experiments to elucidate the functions of normal MECs, including cell adhesion, morphogenesis, and complex signal transduction cascades. They express luminal and stem cell-like markers and present a unique expression profile of epithelial cell markers [23]. When cultured in 3D matrix such as Matrigel, MCF-10A cells have the unique ability to form 3D acinar structures. MCF-10A has been used in the study of benign breast diseases, including investigations into the cellular and molecular mechanisms underlying danazol treatment for fibrocystic breast disease [24]. Although originating from non-neoplastic tissue, MCF-10A cells provide a plastic template for studying the formation of breast tumors. When combined with carcinogenic factors or genetic engineering, they help explore the molecular origins and evolution of breast cancer, and serve as valuable tools for assessing potential therapeutic strategies [25]. The MCF-10 sublines are a series of breast cancer cell lines originating from the MCF-10A cell line [26]. These cell lines provide a stepwise model with the same genetic background for tracking the molecular transition of normal mammary epithelium to fully metastatic invasive ductal carcinoma [27,28].

**Fig. 2.**
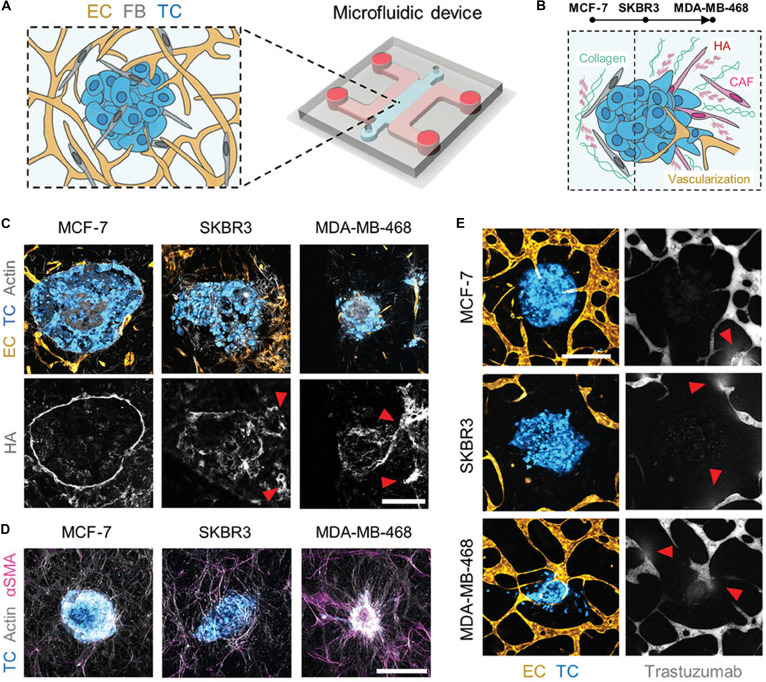
(A) Schematic of tumoroids in microvascular network devices. FB, fibroblast; TC, tumor cell. (B) Schematic of desmoplastic progression in TMEs from MCF-7 to MDA-MB-468. (C) Hyaluronic acid (HA) localization in TMEs (white) and HA traces left by tumor cell migration (red arrows). Scale bar, 200 μm. (D) αSMA (smooth muscle actin) staining of FBs in TMEs. Scale bar, 500 μm. (E) Focal leaks of fluorescent trastuzumab (arrows) in tumoroid microvascular networks. Scale bar, 400 μm [29]. Copyright 2024, Wiley.

Breast cancer cell lines have diverse applications in breast cancer research, providing essential experimental models for understanding the biological characteristics, developmental mechanisms, drug screening, and therapeutic strategies related to this complex disease. Currently, numerous breast cancer cell lines have been established, including MCF-7, SKBR3, and the MDA-MB series. These cell lines exhibit marked differences in genotype, phenotype, and biological properties, making them suitable for various research purposes [29,30]. MCF-7 is the first established and most frequently used model for estrogen receptor-positive breast cancer. The SKBR3 cell line is a well-established model for human epidermal growth factor receptor 2 (HER2)-amplified breast cancer. MDA-MB-231 and MDA-MB-468, which lack expression of estrogen receptor, progesterone receptor, and HER2, are classic models of triple-negative breast cancer. Building upon the characterization of these lines, researchers have cocultured MCF-7, SKBR3, and MDA-MB-468 with endothelial cells (ECs) and fibroblasts to model the desmoplastic tumor microenvironment (TME) across distinct molecular subtypes (Fig. 2) [29].

The sustained proliferative potential of cell lines ensures a stable and abundant source of cells, thereby supporting the reproducibility of experiments. However, prolonged culture can gradually erode key phenotypic and molecular hallmarks that the cell lines once shared with their parental tumor cells. In particular, tumor cell lines commonly exhibit a high degree of genomic instability, which can be exacerbated with increasing passage numbers [31]. Such instability may lead to genetic mutations, phenotypic changes, and functional impairment, resulting in research outcomes that fail to reflect in vivo results. Additionally, cell lines alone can misrepresent primary-tissue complexity [32]. To bolster validity, cell line-based in vitro results should be cross-validated with patient-derived organoids (PDOs), appropriate animal models, and clinical specimens.

### Mammary gland organoids

Sophisticated 3D cell culture systems have emerged as indispensable research tools in epithelial biology, as they uniquely mirror the complexity of native organ microenvironments by faithfully replicating key cellular and extracellular interactions. Immortalized lines, primary cells, or stem cells can be induced to form 3D spheroids that provide a physiologically superior model than their 2D counterparts. However, ordinary cultured cell spheroids have a simple structure and limited functions. In contrast, mammary gland organoids (MGOs) represent more sophisticated and functionally advanced products of 3D culture systems. When embedded in an ECM hydrogel and stimulated with specific cytokines, MGOs demonstrate the ability to differentiate into both luminal epithelial and myoepithelial cells and to self-organize into structures with clear apical–basal polarity. Based on cellular origin, MGOs are broadly classified into 2 categories: those derived from mammary stem cells or progenitor cells and those differentiated from iPSCs.

The prevailing protocol for generating MGOs from mammary stem and progenitor cells relies on microdissection or mechanical-enzymatic digestion of human mammary epithelial fragments, followed by embedding into reconstituted ECM. MGOs obtained from reduction mammoplasty, prophylactic mastectomy, biopsy, or tumor specimens have provided important insights into human mammary biology. Organoids from normal mammary gland tissue have been used to study mammary branching morphogenesis, gland development, and lactation, whereas those from tumor tissue enable the study of cancer initiation, invasion effects, drug screening, and personalized treatment [33–36]. Currently, patient-derived xenografts (PDXs) serve as the gold standard for both basic and translational breast cancer research [37,38]. Nevertheless, the PDX model presents ethical challenges and is high-demand in cost, time, and labor [39]. Consequently, patient-derived MGOs have been considered a highly promising alternative model. Furthermore, gene-editing technology-mediated mutant organoids derived from normal cells can also model breast cancer [40]. Recently, Yuan et al. [41] conducted a relatively comprehensive study on MGOs derived from mouse mammary glands, exploring mammary branching, reproductive cycles, transplantation, regeneration, and modeling breast cancer initiation. Additionally, MGOs derived from stem and progenitor cells isolated from dairy species have been successfully established for studying lactation mechanisms [42,43].

Despite recent advancements, individual tissue variability and limited tissue availability have hindered the widespread application of MGOs derived from primary tissues. iPSCs offer an alternative source, as they can be generated from various somatic cells and differentiate into target cells [22,44]. iPSC-derived organoids are generated through a multi-step, stage-wise differentiation protocol that recapitulates embryonic development, beginning with germ-layer specification and followed by tissue-specific patterning and maturation [45]. In 2017, Qu et al. [46] first induced iPSCs to construct MGOs in vitro, which expressed both luminal and basal markers and produced milk proteins. iPSC-derived MGOs not only recapitulate mammary architecture and function but also circumvent the need for primary tissue, reducing individual variability and ethical concerns.

Another notable example is the D492 mammary epithelial progenitor cell line, which exhibits stem-cell-like potency to yield luminal and myoepithelial progeny that self-assemble into structures resembling terminal duct lobular units, thus providing a robust in vitro model for studying mammary branching morphogenesis and glandular development [47,48]. In summary, MGOs derived from mammary stem cells retain the donor’s genetic background, enabling rapid recapitulation of individualized microenvironments; those generated from iPSCs offer limitless expansion and multilineage differentiation potential that can be directed toward the mammary lineage; meanwhile, the D492 immortalized cell line delivers an easy-to-manipulate, primary-tissue-independent complement model. Together, these 3 sources form a comprehensive in vitro platform spanning physiological studies, disease modeling, and high-throughput applications.

### Coculture of multiple cells

**Fig. 3.**
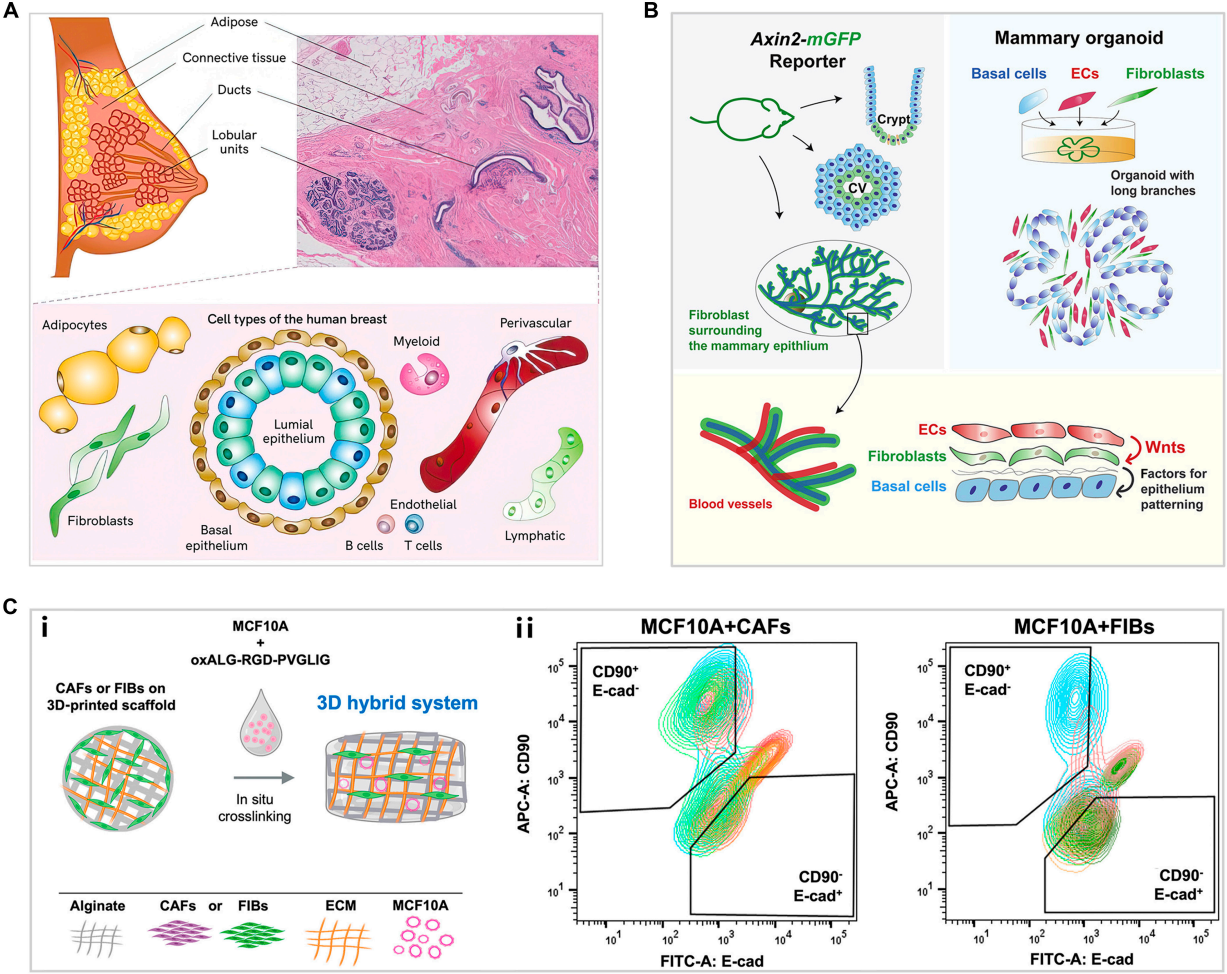
(A) Schematic overview of the key mammary cell types [49]. Copyright 2023, Springer Nature. (B) Illustration of EC-origin Wnts activating fibroblasts in coculture to orchestrate mammary epithelial patterning [52]. Copyright 2021, Elsevier. (C) (i) Schematic representation of the 3D hybrid system composed of MCF-10A with cancer-associated fibroblasts (CAFs) or normal fibroblasts (FIBs). (ii) Sorting of FIB/CAF (CD90^+^/E-cad^−^) and MCF-10A (E-cad^+^/CD90^−^) from coculture systems by flow cytometry [55]. Copyright 2024, Elsevier.

Mammary gland development is profoundly shaped by its surrounding microenvironment. MECs are embedded in a stromal microenvironment composed of various stromal cells, such as adipocytes, fibroblasts, ECs, and immune cells (Fig. 3A) [49]. These stromal cells play a crucial role in regulating tissue structure and the function of epithelial cells. To investigate the role of epithelial–stromal interactions in mammary morphogenesis and tumorigenesis, it is necessary to coculture stromal cells with MECs. Although Transwell coculture models provide useful tools for some studies, incorporating stromal cells into 3D MEC culture offers a more physiologically relevant mammary gland model.

Fibroblasts are responsible for synthesizing various growth factors, proteases, and ECM components, thus affecting mammary development through paracrine signaling, ECM remodeling, and mechanosensing mechanisms. It has been verified that fibroblasts serve as master regulators of mammary branching morphogenesis [41,50]. Sumbal and Koledova [51] investigated fibroblast-mediated fibroblast growth factor (FGF) signaling pathway in mammary epithelial branching morphogenesis through 3D coculture with MGOs. Wang et al. [52] establish a coculture system of ECs, fibroblasts, and MGOs, providing the first evidence that ECs activate Wnt signaling in fibroblasts to regulate epithelial patterning within the mammary gland (Fig. 3B). Adipocytes, which make up a substantial portion of the mammary stroma, are considered a key additional source of milk-producing cells through reversible adipocyte-to-epithelium transdifferentiation. Researchers have simulated pregnancy conditions to study adipocyte transdifferentiation into MECs in coculture with MGOs [53]. This is consistent with earlier findings showing adipocyte-to-epithelial transformation induced by conditioned medium derived from MECs [54].

The occurrence and development of breast cancer involve dynamic changes within a multicellular microenvironment. Stromal–epithelial interactions underpin mammary development and homeostasis but, if disrupted during tumorigenesis, can drive disease progression. Studies using direct coculture models have elucidated the tumor-promoting functions of distinct stromal cell types. To understand how stromal cells initiate transformation, CAFs were cocultured with normal MECs, revealing their ability to produce a matrix that induces malignant transformation of epithelial cells (Fig. 3C) [55]. Under obese conditions, adipose stromal cells have been shown to promote collagen deformation and tumor cell invasion when cocultured with MCF-10A-derived tumor cells [56]. Additionally, direct coculture with polarized tumor-associated macrophages has revealed unique phenotypes and signaling pathways critical for tumor progression that are absent in monoculture or indirect coculture models [57]. Collectively, these studies highlight the multifaceted roles of various cell types, emphasizing the importance of exploring cell–cell interactions for understanding cancer progression.

## Matrix Sources for Mammary Gland Models

Mammary ECM comprises 2 main components: the basement membrane, located directly adjacent to MECs, and the interstitial matrix, situated beyond the basement membrane. The basement membrane is primarily synthesized by luminal epithelial and myoepithelial cells, whereas the interstitial matrix is produced by fibroblasts, adipocytes, ECs, and immune cells. Together, these ECM components not only provide structural support for mammary tissue but also act as key regulators of fundamental processes such as cell growth, differentiation, migration, and apoptosis [58]. The ECM is of great importance for branching morphogenesis and overall development of the mammary gland. Moreover, it is a key driver in the initiation and progression of breast tumors. Therefore, in vitro models require suitable ECM substitutes as scaffolding matrix to create an appropriate microenvironment for cell culture. These ECM substitutes include natural matrices, synthesized matrices, and dECM.

### Natural matrices

Natural matrices, including collagen, fibrin, alginate, chitosan, and other proteins or polysaccharides, play important roles in 3D in vitro cell culture. A variety of hydrogels have been proven to help MECs establish apical–basal polarity. The most direct approach involves providing key basement membrane cues, for example, by using laminin-coated surfaces to guide polarity on breast chips [59]. A more advanced tactic is to architecturally mimic the native tissue environment, as demonstrated by the use of a collagen I and Matrigel bilayer to create a microenvironment that guides MCF-10A cells to form a polarized luminal monolayer [60]. Spatial modifications of collagen I can also alter its physical properties, enabling MECs to form stable mammary ducts even without basement membrane components [61].

**Fig. 4.**
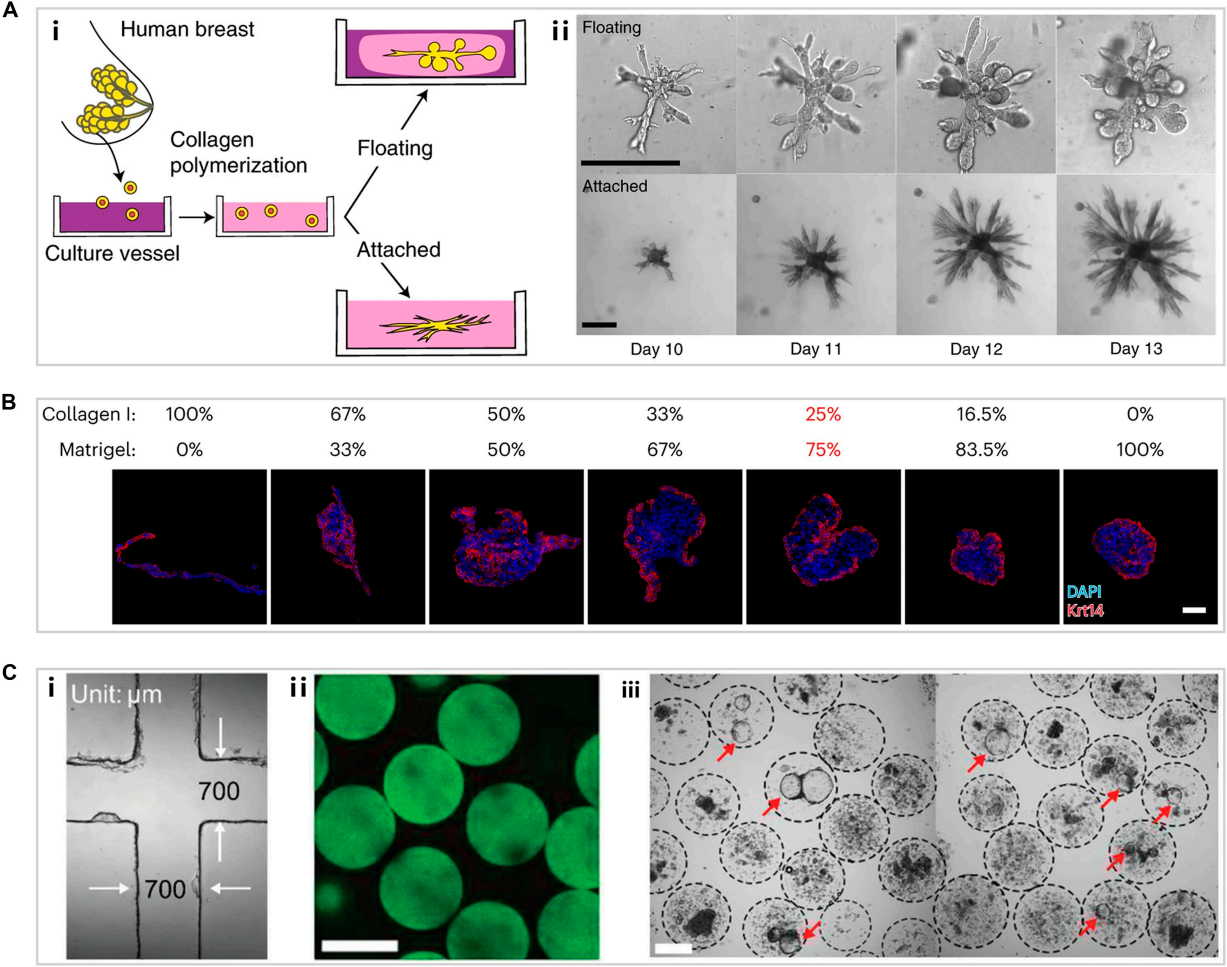
(A) (i) Schematic illustration and (ii) images of primary cells from reduction mammoplasty seeded in floating and attached collagen I gels. Scale bars, 100 μm [67]. Copyright 2021, Springer Nature. (B) Keratin 14 immunofluorescence reveals polarity of MGOs cultured in varying Matrigel/collagen I ratios. Scale bar, 50 μm [41]. Copyright 2023, Springer Nature. (C) (i) Microfluidic chip channel. (ii) Fluorescent alginate microbeads with a uniform size distribution. Scale bar, 400 μm. (iii) Scalable generation of mammary tumor organoids in alginate microbeads. Scale bar, 200 μm [68]. Copyright 2021, Wiley.

For MGO culture, the matrix must closely recapitulate the native mammary gland niche to yield physiologically relevant models. Stable culture conditions using Matrigel-based 3D matrices supplemented with defined growth factors have been successfully established for the cultivation of various organoids, including small intestine, liver, kidney, brain, and mammary. MGOs formed in Matrigel typically adopt a bilayered cystic structure and lose their branching architecture. Although growth factor supplementation can initiate budding, it is insufficient to drive full branching events, as elongated epithelial buds often migrate without myoepithelial coverage [62]. The mechanical properties of the ECM are crucial for branching morphogenesis of MGOs, influencing cell migration and polarity establishment [63]. In the mammary stromal fat pad, high levels of collagen I guide the oriented development of mammary epithelium during puberty [64]. Studies have demonstrated that the branching extension of MGOs is regulated by the mechanical plasticity of collagen in the ECM [65,66]. In floating collagen I gels, organoids form rounded alveoli, whereas in attached gels, surface tension induces cylindrical branching patterns (Fig. 4A) [67]. Notably, neither pure collagen I nor Matrigel alone effectively supports polarity. In contrast, an optimized mixture of collagen I and Matrigel has been shown to better promote branching and polarity (Fig. 4B) [41,62].

The scalability and uniformity of organoid cultures are establishing a standardized platform for translational research, particularly in high-throughput drug screening and personalized medicine development. However, traditional matrices like Matrigel are limited in high-throughput applications owing to their temperature-dependent gelation and batch-to-batch variability. Among natural polymers, alginate is particularly attractive for high-throughput cell encapsulation because of its advantages in biocompatibility and gelation properties in the presence of divalent cations. For example, Fang et al. [68] encapsulated mammary tumor fragments within alginate microbeads using a microfluidic system, achieving mass production of mammary tumor organoids (Fig. 4C). Various biological analyses confirmed that the established organoids preserved key characteristics closely matching the original tumor tissue. Moreover, the generated organoids were subjected to drug sensitivity analysis on microwell arrays, thereby providing an efficient platform for drug screening. A recent study demonstrated that the drug resistance of alginate gel-confined tumor spheroids was 2-fold higher than that of unconfined tumor spheroids, revealing the potential of hydrogel matrices that mimic the ECM to elucidate the relationship between interfacial stress and drug resistance [69].

### Synthetic matrices

The limited mechanical properties of natural polymers have driven the development of synthetic alternatives with superior tunability and well-defined characteristics for MEC culture. Commonly used synthetic polymers, such as poly(lactic-co-glycolic acid) (PLGA), polyethylene glycol (PEG), and polyisocyanopeptide (PIC), support 3D growth by providing customizable microenvironments. Specifically, the well-characterized biodegradability and biocompatibility of PLGA enable its use as an ideal cell transplantation vehicle for tumor formation in vivo [70]. PEG is a bio-inert yet mechanically highly tunable hydrogel, whose bioactivity can be enhanced by supplementing natural matrices to provide bioactive sites. PEG hydrogels engineered to present cell adhesion peptides can be formulated into bioinks with tunable stiffness for the 3D bioprinting of PDOs [71]. This approach has verified that matrix stiffness dictates organoid viability. Furthermore, to enhance cell–matrix interactions, PIC hydrogels functionalized with RGD peptides provide crucial cell adhesion motifs, demonstrating superior biomimicry that supports the efficient and reproducible formation of MGOs from primary tissues [72]. Collectively, the rational design of these synthetic systems addresses distinct limitations of natural matrices, offering controlled microenvironments to dissect tumor biology and therapeutic responses.

In addition to chemical polymers, self-assembling peptide hydrogels (SAPHs) represent a highly promising synthetic scaffold for 3D culture. Tuning the peptide sequence confers precise control over hydrogel properties like charge and stiffness, facilitating customization for specific applications. Researchers have evaluated the efficacy of differentially charged PeptiGel SAPHs in modeling mammary epithelial niche [73]. The positively charged PeptiGel Alpha4 supported MEC viability but failed to support polarized acinus formation, whereas laminin-111-functionalized negatively charged Alpha7 could promote complex MEC acinar morphogenesis. These findings highlight the potential of synthetic polymers as a versatile platform for 3D cell culture, offering tunable properties to support tissue-specific organoid development.

### dECM

Although a variety of natural or synthetic matrices are available to provide 3D culture environments, they still fail to fully recapitulate the tissue-specific structural, mechanical, and biochemical cues present in in vivo models. dECM has emerged as a promising alternative for constructing in vitro models. Derived from native tissues with cellular components removed, dECM preserves the biochemical composition of the ECM while reducing immunogenicity. Decellularization can be achieved through physical (e.g., stirring, ultrasonication, pressurization, and freeze–thaw cycles), chemical (e.g., detergents and acid-based solution treatment), and enzymatic (e.g., proteases such as trypsin, nucleases, and chelating agents) methods. For mammary model construction, the most common dECMs are derived from mammary and adipose tissue [74,75]. While tissue/organ-derived dECM offers physiological relevance, its supply is limited. An alternative is cell-derived ECM, obtained by culturing cells in vitro and subsequent decellularization, which offers greater accessibility, improved consistency, and enhanced biosafety [76].

**Fig. 5.**
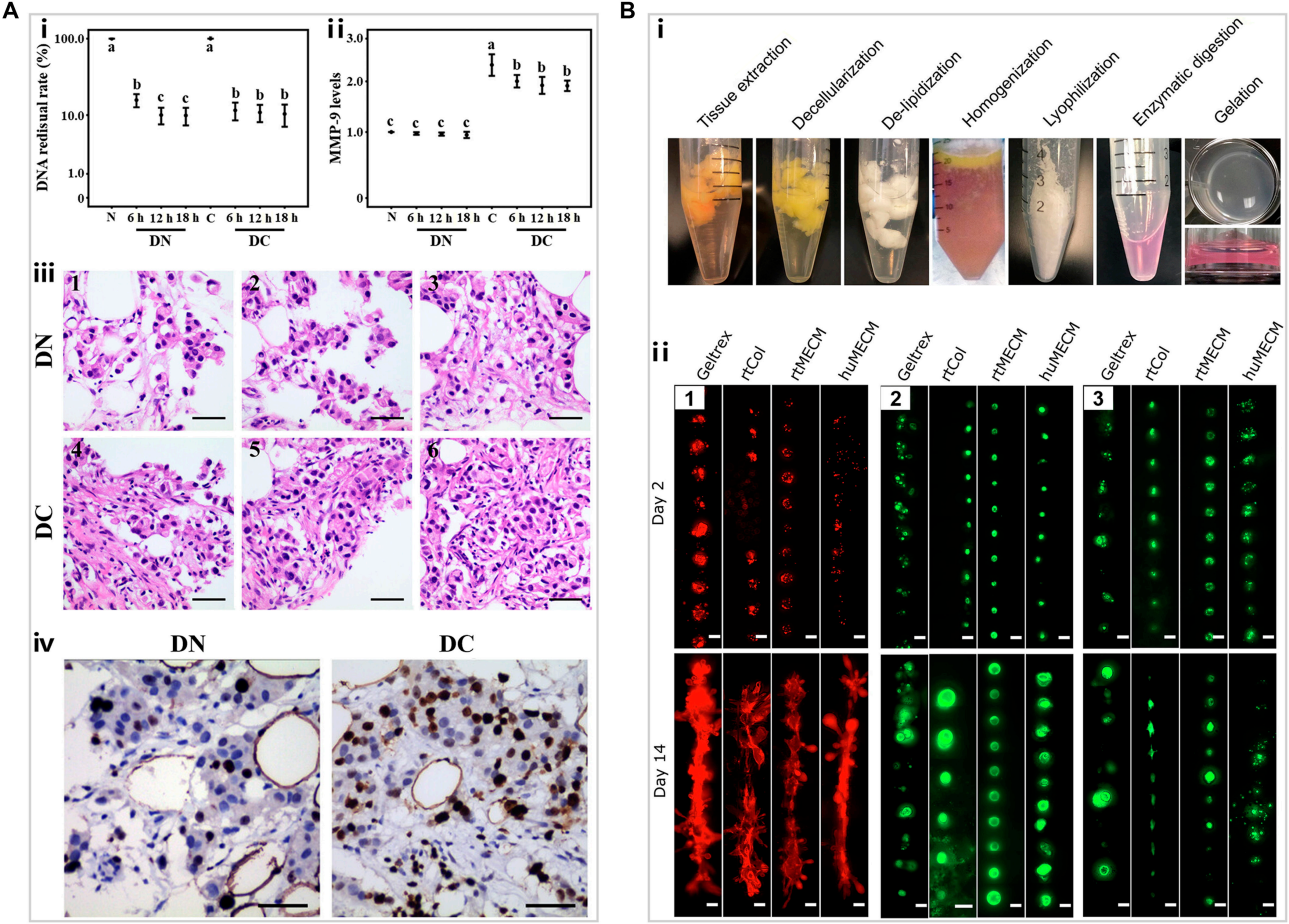
(A) (i) Analysis of DNA rate and (ii) MMP-9 level in fresh normal (N) and cancer (C) tissue samples, as well as decellularized normal (DN) and cancer (DC) tissue samples. (iii) Hematoxylin and eosin (H&E) staining of recellularized DN (1 to 3) and DC (4 to 6) harvested after 10, 20, or 30 d of culture. Scale bars, 50 μm. (iv) Immunohistochemistry staining of Ki-67. Scale bars, 50 μm [78]. Copyright 2018, Wiley. (B) (i) Overview images showing the decellularized hydrogel preparation workflow from human and rat mammary tissues. (ii) MCF-12A cells (1), MCF-7 cells (2) and MDA-MB-468 cells (3) printed in linear patterns within various hydrogels. Scale bars, 200 μm [79]. Copyright 2019, Elsevier.

Decellularized scaffolds can be directly seeded with cells. For instance, Wishart et al. [77] reseeded cells onto dECM scaffolds from tumor-bearing and obese mammary glands to study the role of ECM in tumor metastasis. Of note, the ECM composition of tumor tissues may differ from that of normal tissues. The study by Jin et al. [78] demonstrated a marked increase in matrix mettaloproteinase-9 (MMP-9) expression in tumor samples compared with normal controls (Fig. 5A, i and ii). The decellularized scaffold from cancer tissue supports MCF-7 cell proliferation in vitro, while the decellularized scaffold from normal tissue promotes the migration of MCF-7 cells but inhibits proliferation (Fig. 5A, iii and iv) [78]. This finding suggests that tumor tissue-derived dECM may be more suitable for in vitro tumor modeling to study cancer biology and therapeutic strategies. A more widespread application of dECM involves processing it into hydrogels through dissolution and subsequent gelation under optimal temperature and pH conditions (Fig. 5B, i) [79]. Compared with decellularized scaffolds, decellularized hydrogels demonstrate remarkable advantages in both performance and applicability, notably including tunable pore size, tailorable mechanical properties, and injectability, which make them highly suitable for advanced applications such as bioprinting [75,80,81]. Mollica et al. [79] constructed large 3D bioprinted tumor organoids using human or rat mammary-derived ECM hydrogels (Fig. 5B, ii). Additionally, decellularized adipose tissue hydrogel is used as a bioink to high-throughput print 3D microtumor models in 96-well plates for drug screening [82]. Thus, dECM-based scaffolds and hydrogels offer complementary and physiologically faithful platforms for advancing breast cancer modeling and personalized therapeutic screening.

## Breast-on-a-Chip

Organ-on-a-chip is an advanced biomedical platform that mimics human organ microenvironments on microfluidic devices to replicate key organ functions [83]. By integrating biology, engineering, and materials science, this technology enables the construction of highly biomimetic microphysiological systems in vitro. Recent advances in microfabrication technologies, such as 3D printing and soft lithography, have greatly propelled the development of mammary gland-on-a-chip platforms. These techniques allow precise fabrication of complex biomimetic structures, including microchannels, microwells, and micropores, enabling microscale manipulation of MECs and stromal cells. Consequently, such a technique provides new tools for simulating and studying the tumor niche, cell interactions, metastasis, and drug efficacy [84]. Moreover, compared with traditional 2D cell cultures and animal models, organ-on-a-chip systems can more accurately recapitulate dynamic physiological processes in human organs, reducing reliance on animal experiments and lowering research costs [85,86].

**Fig. 6.**
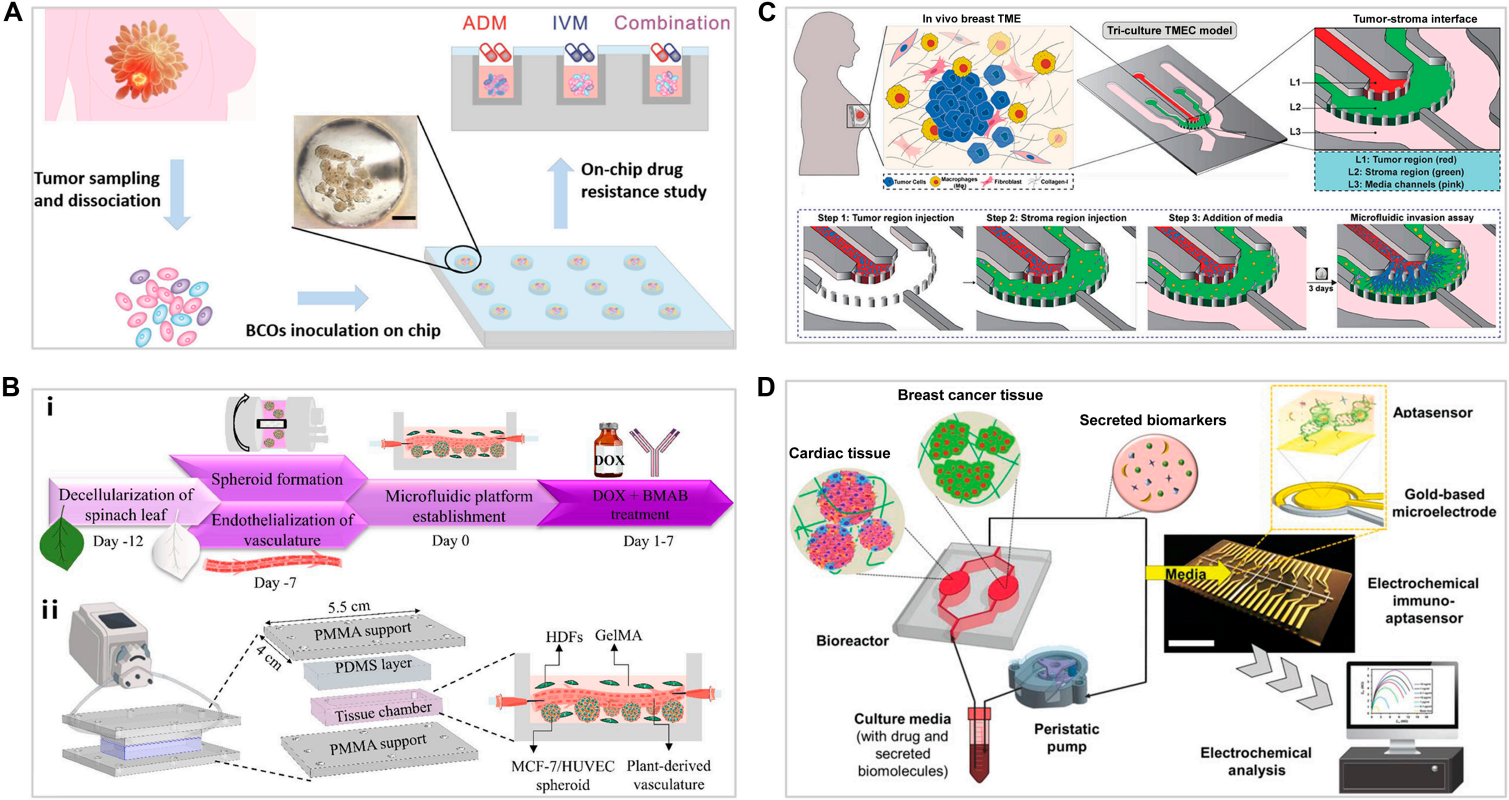
(A) Generation of breast cancer PDOs and on-chip drug resistance analysis [88]. Copyright 2024, American Chemical Society. (B) (i) Workflow for the vasculature-on-a-chip establishment. (ii) Chip layout and tissue-chamber design that models breast cancer [89]. Copyright 2023, Elsevier. (C) Schematic of the microfluidic breast TME-on-a-chip model with tumor region (red), stroma region (green), and side media channels [90]. Copyright 2025, Wiley. (D) Schematic of the integrated cardiac-breast cancer-on-a-chip platform with an electrochemical immuno-aptasensing system. Scale bars, 2 cm [95]. Copyright 2020, Wiley.

Microfluidic technology, a core advantage of organ-on-a-chip systems, allows for dynamic regulation of the cell culture environment, including the application of physiologically relevant mechanical stresses or shear forces, as well as the establishment of hormone or drug concentration gradients to study their effects on cell behavior [87]. High-throughput drug screening is an important application of organ-on-a-chip systems. By culturing multiple tumor organoids derived from the same biological source in parallel within a microwell array and exposing them to different drug treatments simultaneously, researchers can rapidly evaluate drug efficacy and toxicity (Fig. 6A) [88]. This high-throughput screening method not only saves time and resources but also provides more accurate data on drug responses. Since chemotherapeutic drugs are generally transported and diffuse to the tumor site via blood vessels, incorporating vascular networks into drug screening chips is essential for accurately assessing the efficacy of chemotherapy. For example, a vascularized chip was engineered by seeding human umbilical vein endothelial cells (HUVECs) into decellularized spinach leaf veins, thus simulating intravenous drug administration (Fig. 6B) [89].

Furthermore, organ-on-a-chip platforms can be designed as multi-chamber systems to spatially distribute and dynamically model the interactions between parenchymal cells and stromal cells or between mammary tissue and other tissues. Such systems offer more physiologically relevant models than the traditional Transwell system for investigating the mechanisms of breast cancer invasion and drug treatment responses. Chip platforms excel at deconstructing the complex crosstalk within the local TME. For instance, designed to achieve spatially partitioned but interactive coculture, a dedicated TME chip was employed to dissect the driving role of coordinated stromal–immune crosstalk in breast cancer progression (Fig. 6C) [90]. Beyond modeling the primary site, spatial control of organ-on-a-chip technology is pivotal for reconstructing the path to distant metastasis. Given that the bone is a preferred site for breast cancer metastasis [91,92], a 3D bioprinted model that reconstructs the bone metastatic niche was developed, providing insights into how distant microenvironments influence tumor fate [93]. A critical consideration in breast cancer therapy is chemotherapy-induced cardiotoxicity, a major adverse effect that can lead to ventricular dysfunction or clinical heart failure, markedly impacting patient outcomes [94]. A heart–breast cancer chip platform epitomizes this by integrating functional cardiac tissue to monitor the adverse cardiotoxic effects of chemotherapeutic agents (Fig. 6D) [95]. This interconnected system not only models a major clinical complication but also enables the parallel analysis of secreted biomarkers, offering a comprehensive tool for therapeutic safety assessment. Collectively, these chips transcend simplified models to capture the interconnected biology of cancer progression and treatment, collectively highlighting the advancing impact of organ-on-a-chip technology.

## Biomedical Applications

### Mammary gland development

The mammary gland develops through a highly dynamic, multi-stage process beginning postnatally and encompassing puberty, pregnancy, lactation, and involution [96]. This development involves complex mechanisms of cell differentiation, tissue remodeling, and hormonal regulation [97]. During puberty, hormones and growth factors jointly regulate the branching morphogenesis of mammary ducts. Pregnancy triggers extensive proliferation of epithelial cells and alveolar development under hormonal stimulation. In the lactation phase, luminal epithelial cells and basal cells carry out milk synthesis and ejection under the action of prolactin and oxytocin [98]. After weaning, the mammary gland enters the involution phase, characterized by programmed cell death, tissue remodeling, and the redifferentiation of fat cells. These stages are crucial not only for women’s psychological and reproductive health but also for providing nutrition and immune protection to newborns.

In recent years, MGO has emerged as a powerful in vitro model for investigating mammary gland development across reproductive cycles. For instance, Sumbal et al. [99] established a mouse MGO model that simulated both lactation and involution, successfully reproducing the gland’s morphological and functional changes during lactation. Critically, oxytocin-responsive contractility of myoepithelial cells has been directly observed in lactating MGOs, confirming their functional maturation within the organoid system. In 2021, Todhunter et al. [100] demonstrated that MGOs cultured in microcontainers exhibit spontaneous, rhythmic myoepithelial contractions—independent of exogenous hormonal stimulation. This phenomenon likely arises from the functional differentiation of myoepithelial cells induced by spatial constraints and endogenous matrix accumulation. While this is an intriguing finding, the study exhibits notable limitations, such as insufficient luminal cell differentiation and impaired ductal elongation. Therefore, this line of research requires further optimization and exploration.

**Fig. 7.**
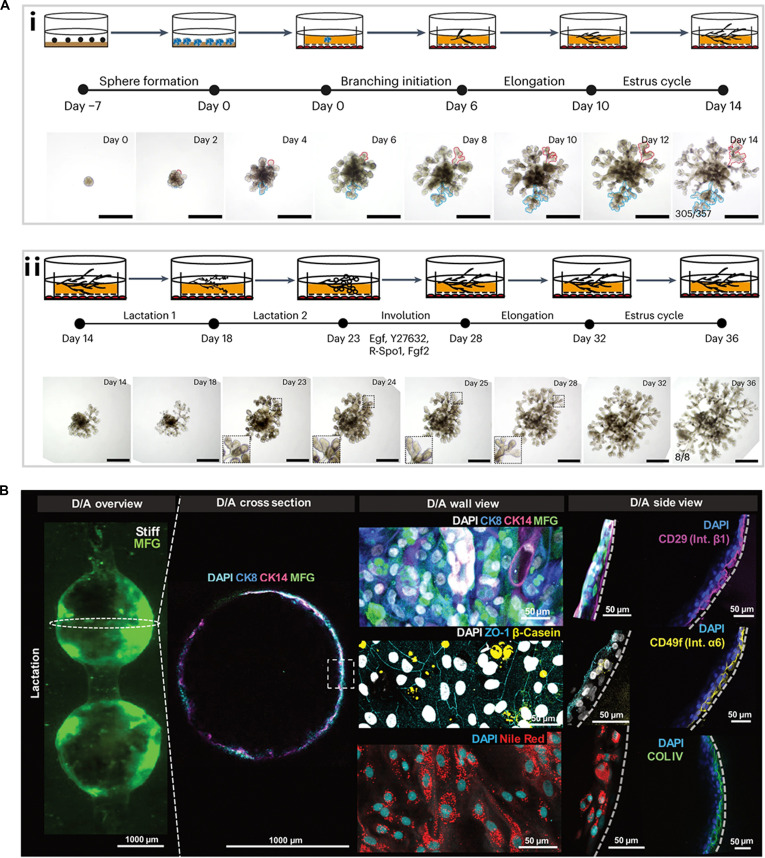
(A) (i) Timeline and morphological changes during mini gland development from sphere formation through the estrus cycle. (ii) Timeline and morphological changes during the mini gland lactation–involution–estrus cycle. Scale bars, 500 μm [41]. Copyright 2023, Springer Nature. (B) Immunofluorescent staining and Nile red staining of MECs at day 7 after seeding [101]. Copyright 2025, The American Association for the Advancement of Science.

Recently, Yuan et al. [41] developed a dynamic MGO culture system that successfully reconstructed mammary structures with cellular heterogeneity and hormonal responsiveness. This organoid culture system recapitulates key aspects of mammary developmental, progressing from initial spheroid formation to dendritic branching, and sequentially undergoing pseudo-estrus cycle and reproductive cycle, with the capacity to reenter a new estrus cycle (Fig. 7A). During the lactation phase, a 2-step lactogenic program drives alveolar formation and milk secretion, thereby elucidating the specific roles of hormones such as progesterone, prolactin, and oxytocin in reproduction. The development of this sophisticated MGO culture system represents a substantial technological advancement, offering a robust tool to dissect the causal relationships underlying mammary morphogenesis and functional differentiation. In addition to organoid models, the 3D-printed mammary duct/alveoli (D/A) model developed by Hasenauer et al. [101] offers novel approaches for investigating the hormonal regulation, nutritional metabolism, and biomechanical factors associated with lactation (Fig. 7B). Together, these advanced in vitro platforms enhance our comprehension of mammary gland development and lactation mechanisms, paving the way for improved maternal and infant health interventions.

### Breast cancer models

Globally, breast cancer is the most prevalent malignancy among women in terms of both incidence and mortality, posing a substantial public health challenge [4,102,103]. Advances in cancer diagnosis and early treatment have markedly enhanced patient prognosis and quality of life [104–106]. However, metastasis, treatment resistance, and tumor recurrence remain the primary obstacles to successful treatment [107,108]. Therefore, deeper insight into oncogenic drivers, TME dynamics, and the mechanisms of recurrence and metastasis is crucial for developing effective therapeutic strategies.

A critical approach to unraveling breast cancer initiation is the study of tumorigenesis starting from normal MECs, which is essential for prevention efforts and identifying risk reduction. Nontumorigenic MECs such as MCF-10A serve as widely used models to evaluate the transforming effects of chemical carcinogens, including benzo[a]pyrene, aflatoxin, and cadmium [109–111]. Environmental polycyclic aromatic hydrocarbons exert their toxicity and carcinogenic responses by binding to aryl hydrocarbon receptors (AhRs) on cells. A model system of engineered cell lines with graded AhR expression was established from an immortalized normal MEC line with low endogenous AhR [112]. This system demonstrated that elevated AhR expression and activity drive the key pro-oncogenic program of epithelial–mesenchymal transition (EMT). EMT is a reversible transcriptional switch whereby epithelial cells lose apical–basal polarity and cell–cell adhesion, acquire mesenchymal cytoskeleton and motility, and gain the capacity to traverse basement membranes [113,114]. This shift is widely recognized as a pivotal mechanism in cancer initiation and metastasis promotion [115–117]. Notably, MCF-10A cells treated with factors like insulin or leptin lose epithelial features, such as cell polarity and intercellular adhesion, while gaining mesenchymal characteristics, such as enhanced cell migration and invasive capacity [118,119]. This transformation is crucial for understanding the mechanisms of cancer invasion and metastasis.

**Fig. 8.**
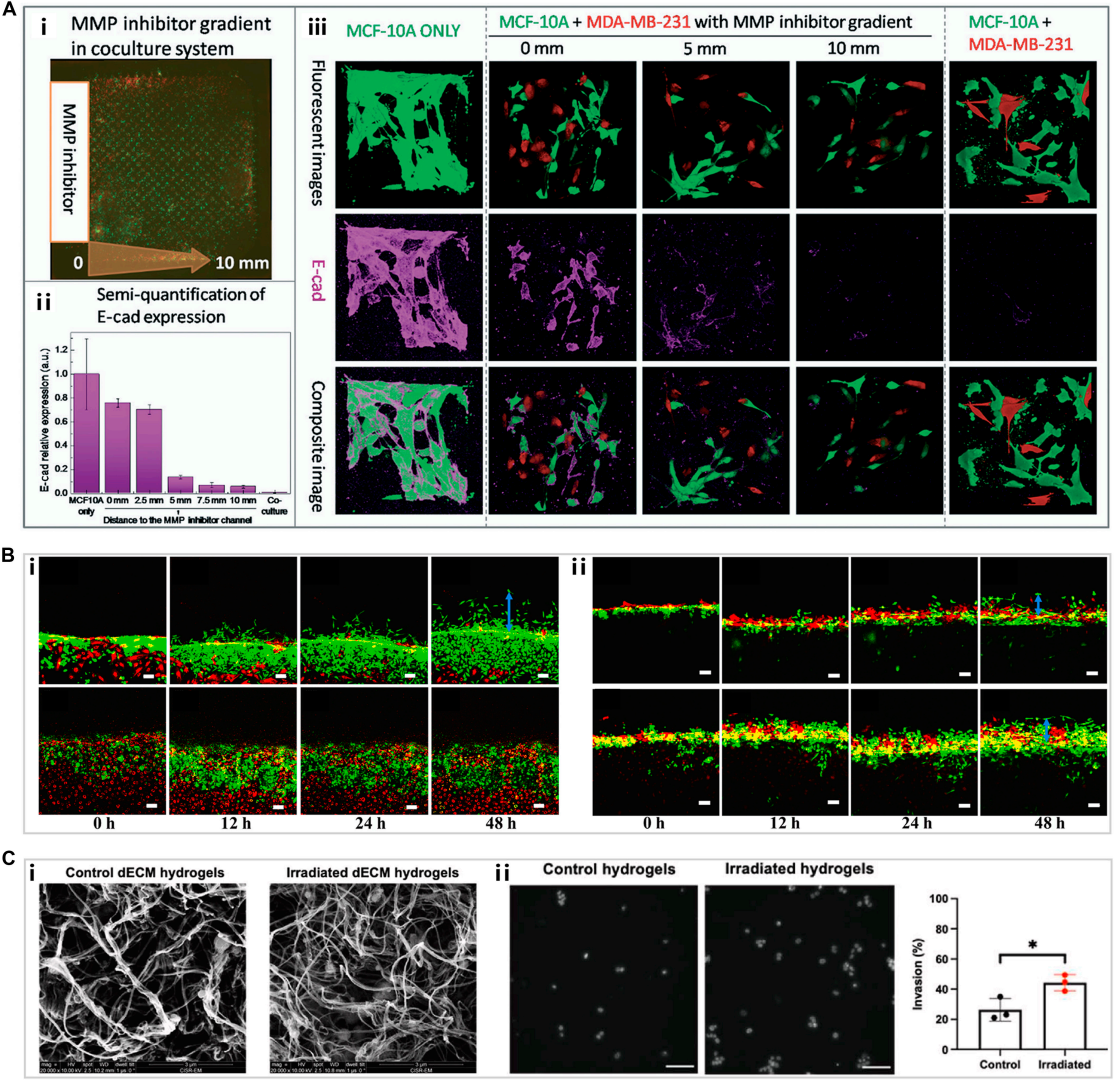
(A) (i) Overview of the microfluidic chip. (ii) Semiquantitative analysis of E-cadherin expression along the increasing distance from the MMP-inhibitor channel. (iii) Representative images of the cells’ morphology and E-cadherin expression within individual microchambers [120]. Copyright 2017, Royal Society of Chemistry. (B) (i) Extravasation of MDA-MB-231 (upper panel) and MCF-7 (lower panel) at indicated time points. Scale bars, 100 μm. (ii) Extravasation of MDA-MB-231 over a time course of 48 h after tocilizumab (upper panel) and reparixin (lower panel) treatment. Scale bars, 100 μm [125]. Copyright 2024, Elsevier. (C) (i) Comparison of hydrogel architecture by scanning electron microscopy (SEM) analysis. (ii) Cell nuclei quantification of invading 4T1 cells encapsulated in 2 groups of hydrogels. Scale bars, 100 μm [128]. Copyright 2024, Elsevier.

A recent study developed a 3D bio-microfluidic system to simulate the heterogeneous tumor microstructures in vivo and to reveal the mechanisms underlying tumor cell invasion [120]. When invasive MDA-MB-231 cells were cocultured with MCF-10A cells, the tubular structures formed by MCF-10A cells were disrupted. This disruption was attributed to the down-regulation of E-cadherin in MCF-10A by MDA-MB-231, facilitating the breakdown of epithelial architecture and promoting invasion and metastasis (Fig. 8A) [120]. These findings corroborate previous studies highlighting the critical role of E-cadherin loss in tumor invasion [121]. Tumor cell extravasation refers to the process by which tumor cells pass through the endothelial layer of blood or lymphatic vessels to enter the surrounding tissue, which is a key step in the process of tumor metastasis [122–124]. The extravasation process involves a variety of biological mechanisms, including the interaction between tumor cells and ECs, ECM degradation, and enhancement of cell motility. To better understand these dynamics, Qian et al. [125] constructed a tumor chip featuring a vascular channel that allows real-time visualization of cell interactions during extravasation and the study of underlying molecular mechanisms. Their results demonstrated that MDA-MB-231 cells could penetrate the endothelial barrier and invade the hydrogel within 12 h, exhibiting markedly greater extravasation ability than MCF-7 cells (Fig. 8B, i). Additionally, drug screening with the chip confirmed the effectiveness and mechanism of tocilizumab and reparixin in inhibiting the extravasation of MDA-MB-231 cells (Fig. 8B, ii).

In addition to cancer cells, the ECM is a fundamental element of the TME and a vital component for in vitro tumor modeling. During breast cancer progression, the mammary ECM undergoes pathological remodeling, characterized by excessive collagen deposition, aberrant cross-linking, and increased matrix stiffness [126]. A research team reported that the dECM derived from CAFs contained overexpressed proteins consistent with those in tumor tissues. Furthermore, this CAF-derived dECM hydrogel induced a malignant phenotype in MECs. This result aligns with the team’s prior findings from a direct CAF-MEC coculture study, together reinforcing the critical role of altered ECM in driving EMT [55,127]. Another team developed a bioprinted breast cancer model using a biomimetic bioink derived from decellularized mammary tissue, which was further supplemented with collagen I to investigate its effects on cancer cells [80]. The results showed that incorporating collagen I enhanced invasive behavior, malignant progression, and chemoresistance of breast cancer cells. Additionally, Zhu et al. [128] used irradiated mouse fat pads to prepare decellularized hydrogels and further explored the changes in ECM after radiotherapy and their impact on breast cancer recurrence. The study indicated that radiotherapy may promote breast cancer recurrence by altering ECM composition and architecture, thereby increasing the invasiveness of tumor cells (Fig. 8C). This discovery provides a new perspective for understanding the mechanisms of tumor recurrence after radiotherapy and offers potential targets for developing targeted therapeutic strategies.

### Drug screening

Reliable evaluation of drugs demands robust efficacy and cytotoxicity assessment before clinical entry. Traditional 2D monolayers lack intercellular signaling and native ECM cues, yielding response rates that frequently mis-predict in vivo efficacy. Cell spheroids, organoids, and micro-tissues have therefore supplanted 2D cultures, restoring gradients of oxygen, nutrients, and metabolites alongside cell–ECM crosstalk that govern proliferation, dormancy, and drug penetration [129]. Notably, advanced biotechnologies such as organ-on-a-chip and 3D bioprinting are driving the development of engineered 3D models with unprecedented physiological complexity. They narrow the divide between in vitro models and complex in vivo systems, accelerating drug discovery and precision medicine.

**Fig. 9.**
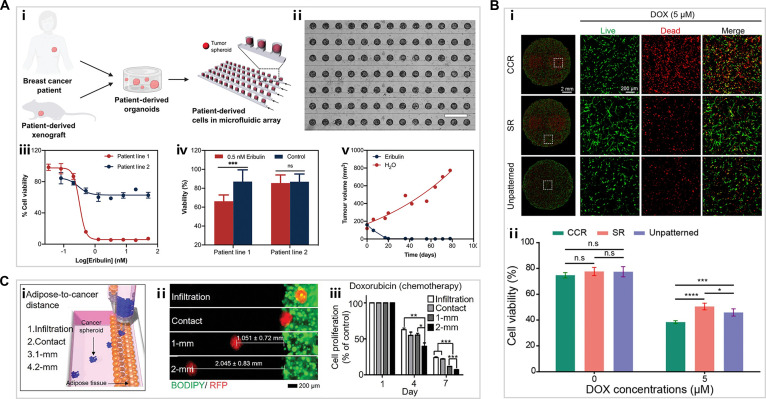
(A) (i) Schematic of the PDO microfluidic array generation strategy. (ii) Representative image showing the arrayed PDOs. Scale bar, 1 mm. (iii) Off-chip eribulin sensitivity of PDOs derived from patients. (iv) Viability of PDO arrays from lines 1 and 2 after 5-d perfusion with 0.5 nM eribulin or drug-free medium (control). (v) In vivo eribulin response of the PDX matching patient line 1 [130]. Copyright 2021, Wiley. (B) (i) Live/dead staining images and (ii) corresponding viability quantitation for cancer cell-rich (CCR), stroma-rich (SR), and unpatterned regions after doxorubicin (DOX) treatment [132]. Copyright 2024, Elsevier. (C) (i) Schematic illustration of printed composite tissue model. (ii) Fluorescence images of bioprinted adipose tissue (green) and MCF-7 spheroids (red) in composite tissue models. (iii) Relative cell proliferation of breast cancer spheroids after 6 d of doxorubicin treatment [134]. Copyright 2024, Wiley.

Organ-on-a-chip platform enables high-throughput cell culture and drug screening. Some microfabricated structures, like microhole or microcolumn arrays within chips, enable mass production of uniform cell spheroids or organoids. For instance, Prince et al. [130] designed a microfluidic chip that is capable of efficiently generating breast cancer PDO arrays (Fig. 9A, i and ii). Notably, the on-chip culture of PDOs yielded drug sensitivity profiles that were highly consistent with the in vivo efficacy of eribulin (Fig. 9A, iii to v) [130]. Ro et al. [131] further advanced such a platform by embedding perfusable vascular channels within a microsphere array, enabling simultaneous modeling of tumor–endothelial interactions and physiologically relevant drug diffusion, thereby supporting integrated studies of tumor biology and drug screening. Building on these proof-of-concept demonstrations, PDO-on-chip technology holds considerable potential to predict clinical efficacy and inform therapeutic decision-making.

3D bioprinting further advances drug screening by offering precise spatial patterning of cell and ECM composition, thereby better recapitulating the complex microenvironment of tumor tissue. Yuan et al. [132] precisely patterned MDA-MB-231 cells, ECs, and CAFs in a biomimetic ECM ink using 3D bioprinting. This model features densely packed cancer cell regions of varying sizes, surrounded by stroma rich in microvasculature, effectively mimicking the pathological changes observed in patient samples. Cells in different regions of the bioprinted TME exhibited distinct drug responses, highlighting spatial heterogeneity in chemosensitivity (Fig. 9B). Adipose tissue constitutes a substantial proportion of breast stromal tissue and has been demonstrated to influence the invasion and drug resistance of breast cancer via paracrine signaling [133]. To investigate these interactions, researchers embedded breast cancer spheroids and adipose tissue at varying distances within a 3D-printed model, demonstrating the model’s ability to accurately assess drug efficacy (Fig. 9C) [134].

Breast tumors are complex ecosystems, with heterogeneity arising not only from diverse cell populations but also from variations in the ECM. Through ECM remodeling, tumor cells establish physical and biochemical barriers that limit both immune cell access and drug delivery, thereby facilitating immune evasion and treatment resistance. Therefore, developing 3D models that can reproduce the complexity of the tumor ECM is of great importance for studying anticancer drugs. In one approach, primary breast cancer samples from patients were decellularized and reseeded with donor cancer cells to study the response to chemotherapy and the risk of disease recurrence. Landberg and colleagues [135] found that cancer cells cultured on patient-derived scaffolds (PDSs) exhibited increased resistance against the frontline chemotherapy drugs than their 2D counterparts. Further investigations revealed that the transcription profiles of cancer cells treated with 5-fluorouracil or doxorubicin varied depending on the specific microenvironment provided by the PDSs [136]. These findings highlight the substantial impact of the TME on treatment outcomes and suggest that scaffold-based models provide a basis for formulating precise treatment plans.

In addition to chemotherapy, cellular immunotherapy and combination immunotherapy are gradually becoming the new standards of oncological treatment. While cellular immunotherapy has been authorized for the clinical treatment of a variety of hematologic malignancies, many challenges remain in applying it to solid tumors such as breast cancer [137,138]. Recently, a sophisticated breast cancer-on-chip platform has been established to recapitulate the entire process—from infusion to recruitment and infiltration—of chimeric antigen receptor (CAR)-T cells attacking tumor aggregates while simultaneously monitoring the ensuing cytokine dynamics [139]. Therefore, in addition to the screening of chemotherapeutic drugs, breast-on-a-chip models hold great potential for monitoring emerging therapies. With sustained investment in translational research, these models could substantially improve patient outcomes and reduce the global burden of breast cancer.

## Conclusion and Outlook

In summary, the development and application of mammary gland models have greatly enhanced our understanding of breast biology and pathology. Cell models, 3D hydrogel-based matrix, and breast-on-a-chip technologies each offer unique advantages in simulating the complex microenvironment of the mammary gland. Cell models provide a fundamental platform for studying the basic mechanisms of breast development and tumor progression; 3D hydrogel-based matrix offers a more physiologically relevant environment by mimicking the ECM, allowing for the investigation of cell–matrix interactions; and breast-on-a-chip technologies integrate multiple physiological components for organ-level function study and drug response evaluation in a highly controlled and dynamic environment. Collectively, these platforms have been instrumental in elucidating the intricate processes of gland development, tumor initiation, and metastasis, as well as in the development and monitoring of therapeutic interventions.

Despite considerable progress, future research in mammary tissue and disease modeling still faces challenges and opportunities. A primary challenge is to further enhance the physiological relevance of mammary models. This requires a paradigm shift from epithelial-centric systems to ones that systematically incorporate the critical stromal and immune components of the native breast microenvironment. Such integration is crucial for accurately modeling complex processes like therapy response. For instance, the efficacy of some chemotherapeutics stems from both direct cytotoxicity and immunomodulation. Therefore, incorporating tumor-immune cocultures into drug screening platforms is essential to better recapitulate the in vivo context and improve predictive accuracy, particularly for evaluating combination immunotherapies. A critical and often overlooked dimension is the profound gender gap in model systems. Although beast diseases are rare in men, they do occur, yet research in this area remains disproportionately scarce. Fundamental anatomical, hormonal, and molecular differences between male and female mammary tissues preclude the direct extrapolation of findings from female-centric models. Consequently, the development of biologically faithful, male-specific mammary models is not only scientifically warranted but also urgently needed to close this persistent knowledge gap and advance precision medicine for men with breast disease. Another priority is developing high-throughput and automated platforms that accelerate large-scale drug screening and advance personalized medicine. The integration of advanced imaging and sensing technologies is also crucial for real-time monitoring of cellular behavior and therapeutic efficacy. These advancements will enable more accurate and efficient preclinical testing of potential therapeutic agents.

Achieving these goals will require interdisciplinary collaboration and innovation. The field of breast modeling is inherently multidisciplinary, involving expertise from cell biology, materials science, bioengineering, and clinical medicine. Continued collaboration across these disciplines will be essential for developing the next generation of breast models capable of more accurately simulating the human breast. Additionally, the integration of computational modeling and machine learning approaches can provide new insights into the complex interactions within the breast microenvironment and help optimize model design. By fostering a collaborative and innovative research environment, we can accelerate the development of more effective breast models and translate these advancements into clinical practice. Beyond medical applications, the potential of in vitro mammary models deserves exploration in broader fields such as food technology. Drawing inspiration from advances in cellular agriculture for cultured meat, engineered mammary models may serve as biofunctional platforms for the sustainable, scalable production of cell-cultured milk. Such systems could offer innovative strategies to supplement conventional dairy supply, address maternal milk shortages, and meet the specific nutritional needs of neonates. It is hoped that through future interdisciplinary collaboration, the challenges in the development of physiology-related models, high-throughput screening platforms, and translational applications can be overcome, thereby providing stronger support for breast-related medical research and contributing to the advancement of human health.
